# Role of microRNA in Endocrine Disruptor-Induced Immunomodulation of Metabolic Health

**DOI:** 10.3390/metabo12111034

**Published:** 2022-10-28

**Authors:** Nitya Shree, Zehuan Ding, Jodi Flaws, Mahua Choudhury

**Affiliations:** 1 Department of Pharmaceutical Sciences, Irma Lerma Rangel College of Pharmacy, Texas A&M University (TAMU), College Station, TX 77843, USA; 2 Department of Comparative Biosciences, University of Illinois Urbana-Champaign, Urbana, IL 61802, USA

**Keywords:** EDCs, DOHaD, innate immune system, metabolic health, miRs

## Abstract

The prevalence of poor metabolic health is growing exponentially worldwide. This condition is associated with complex comorbidities that lead to a compromised quality of life. One of the contributing factors recently gaining attention is exposure to environmental chemicals, such as endocrine-disrupting chemicals (EDCs). Considerable evidence suggests that EDCs can alter the endocrine system through immunomodulation. More concerning, EDC exposure during the fetal development stage has prominent adverse effects later in life, which may pass on to subsequent generations. Although the mechanism of action for this phenomenon is mostly unexplored, recent reports implicate that non-coding RNAs, such as microRNAs (miRs), may play a vital role in this scenario. MiRs are significant contributors in post-transcriptional regulation of gene expression. Studies demonstrating the immunomodulation of EDCs via miRs in metabolic health or towards the Developmental Origins of Health and Disease (DOHaD) Hypothesis are still deficient. The aim of the current review was to focus on studies that demonstrate the impact of EDCs primarily on innate immunity and the potential role of miRs in metabolic health.

## 1. Introduction

Deteriorated metabolic health is rapidly becoming a serious public health burden across all genders, ages, and socioeconomic groups [1]. Currently, obesity is one of the leading causes of death, which impacts 35% of the US population and is predicted to increase to 42% by 2030 [2]. Additionally, medical expenditures for obesity-related conditions are expected to exceed 1 trillion USD by 2025 [3]. It is evident that the effort to treat and prevent obesity still requires improvement. Obesity has been primarily attributed to overnutrition, sedentary lifestyle, and genetic inheritance; however, it is unlikely that these are the only factors responsible for this exponential rise of the epidemic in recent years [4,5]. Emerging evidence suggests that chronic exposure to environmental chemicals may contribute to the rapid rise in the prevalence of metabolic disorders [6,7]. Additionally, disturbances during crucial developmental windows can promote subtle changes in gene expression, leading to modifications in biological and molecular processes. Ultimately, these modifications alter the developmental trajectory, leaving permanent, long-lasting metabolic dysfunction that may persist from adolescence to subsequent generations [8]. The aim of the current review was to recognize the impact of endocrine-disrupting chemicals (EDCs) primarily on innate immunity and the potential role of miRs in metabolic health.

One group of environmental chemicals that has gained widespread attention is EDCs. EDCs are abundantly present in the environment, and they can be found in food packaging, personal care products, and other manufactured products [9]. Each year, new compounds identified as endocrine modulators add to the gravity of further research to be conducted [10]. EDCs are a threat to global human health due to their carcinogenic and mutagenic properties. However, the implications of EDC’s behavior contributing to metabolic health are not fully understood [11]. They can interfere with normal bodily functions by mimicking, blocking, or interfering with the hormonal activity involved in the endocrine system [12]. In addition to these traditional pathways, recent evidence suggests that EDCs can modulate via epigenetic regulations or non-coding RNA mediated pathways [13].

Exposure to EDCs has been proven to alter normal immune processes that are essential for metabolic homeostasis [14]. The unique tissue architecture of adipose and its close proximity to immune cells facilitates their interaction for survival during periods of starvation and immunological challenge [15,16]. This crosstalk between the immune system and adipocytes is one of the key mechanisms in the progression of metabolic disease and any perturbations in this communication can contribute to the immunomodulation of metabolic health [16]. Furthermore, the impact of EDCs on the immune cells and immunity has also been demonstrated [17]. Several reports confirm the effect of these chemicals on the function, development, and lifespan of immune cells [18,19,20]. It has been primarily suggested that EDCs bind to the transporting proteins in the blood and subsequently to the nuclear receptors, thus modifying the permeability of the ion influx and leading to abnormal immune function [19,21,22]. 

EDCs are also known to interfere with the hormone homeostasis and endocrine signaling, both of which directly govern embryonic development during pregnancy [23]. Accumulating evidence reveals that exposure to EDCs in early life can propagate non-communicable diseases throughout one’s lifetime [24]. This is in line with the DOHaD hypothesis, which suggests that exposure to environmental disturbances during pregnancy can lead to offspring adaptations, potentially leading to the development of chronic diseases in the later stages of life [24]. These adaptations are majorly thought to be through epigenetic modifications, including histone modification and DNA methylation [25,26]. In addition to epigenetic modifications, the impact of EDCs on non-coding RNAs (ncRNA) is being studied [27,28,29,30,31,32,33]. However, among all the ncRNA types, miRs have gained interest for their putative regulation by EDCs in metabolic disturbances. 

**Figure 1:**
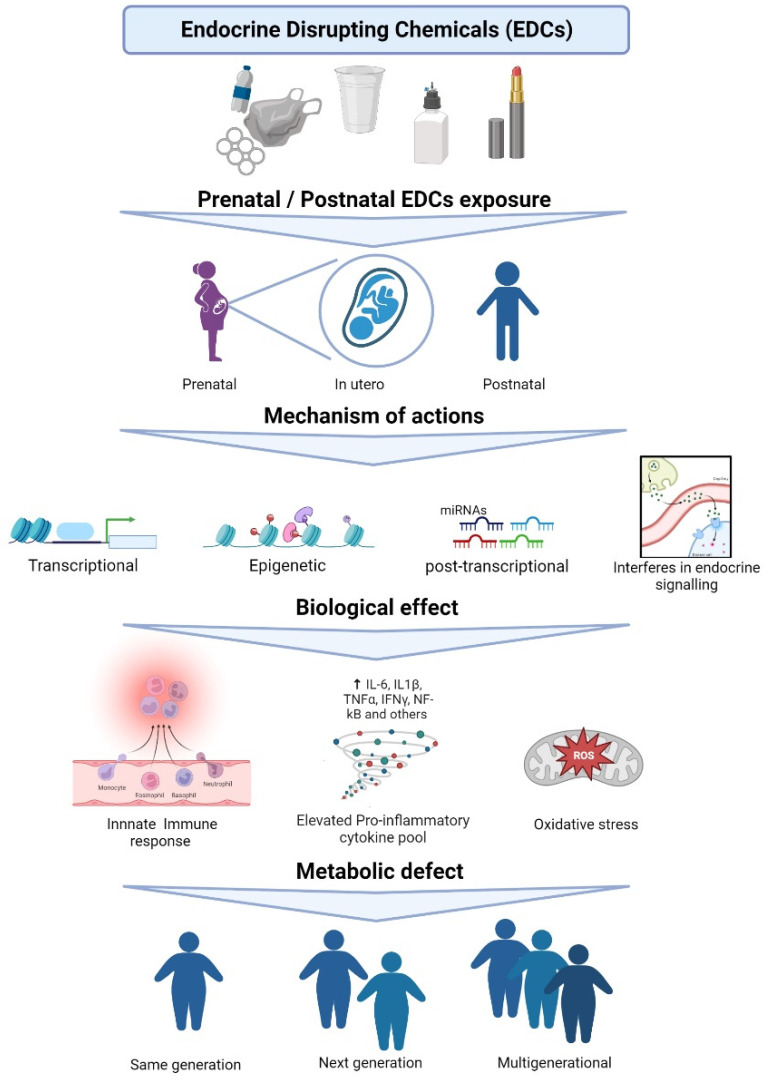
Various mechanisms of actions of EDC leading to metabolic health defects.

In the current review, we highlight the impact of EDCs exposure on immunomodulation via miR regulation in metabolic disease. We discuss the scenario in the same generation, as well as multigenerational and transgenerational changes. From the literature, we also include a list of EDCs with a potential direct or indirect role in the alteration of the immune system and metabolism via miRs. Finally, we propose the link between EDCs, miRs, immune response, and metabolism (Figure 1) to complement the existing knowledge of the detrimental effect of EDCs on immunomodulation of metabolic health.

## 2. EDCs Exposure and the Immune System

The progression of disease due to EDC exposure during development has been associated with alterations in the immune system of humans [34]. The immune system is a collaborative network of cells and proteins that protect the body against anything that is identified as non-self or foreign. It comprises bone marrow, the thymus, the spleen, white blood cells, antibodies, the complement system, and the lymphatic system [8]. The complementary machineries within the immune system are innate immunity and adaptive immunity. The innate immune response is the first line of defense that is activated to destroy intruding material. It is non-specific and acts via physical barriers (e.g., skin and mucous membrane, cilia) and chemical barriers (e.g., lysozyme, gastric juice, and saliva). The second line of defense is the adaptive immune response. It is designed to react against specific recognized antigens located on foreign material. It is carried out by the lymphocytes that are responsible for the development of immunological memory. Innate control of adaptive immunity plays a crucial role during the development of immune response, which further contributes to the activation of long-lasting adaptive immunity [35]. Exposure to EDCs can directly affect the innate immune response, contributing to endocrine imbalance [36]. Therefore, we focus on the impact of EDCs on the innate immune system. 

The innate immune system consists of monocytes, macrophages, mast cells, neutrophils, and natural killer cells [8]. Monocytes express various receptors capable of monitoring and sensing environmental changes. Typically, a monocyte cell circulates in the blood for about 1–3 days before migrating into the tissues where they differentiate into a macrophage or a dendritic cell. Monocytes and macrophages are one of the major sources of tumor necrosis factor-alpha (TNF-α) [37]. In a mouse macrophage cell line, EDCs can alter the inflammatory response and the host’s defense mechanism against pathogens [38]. Overall, this demonstrates that alteration in the TNF-α secretion pattern by macrophages at local disease sites is accompanied by the development of inflammatory diseases [39]. A study by Kuan et al. showed elevated levels of TNF-α secretion in RAW 264.7 cells exposed to bisphenol A (BPA), an industrial plasticizer [40]. One of the most recent meta-analyses conducted to investigate the link between EDCs and inflammatory markers in humans revealed that BPA exposure is linked to the differential levels of C-reactive protein (CRP) and IL-6 [41]. Di-2-ethylhexyl phthalate (DEHP) enhances TNF-α production from monocytes/macrophages in vitro and in vivo [39]. Studies have shown that EDCs, including DEHP, BPA, and dichlorodiphenyltrichloroethane (DDT), can modulate the production of several interleukins, such as IL-6, IL-8, IL-4, and IL-1β [42]. This implicates EDCs’ role in metabolic dysregulation because alteration in the inflammatory and non-inflammatory cytokine pools is a common scenario in metabolic disorders [43].

In addition to macrophages and monocytes, mast cells mediate inflammatory responses, such as allergic reactions and hypersensitivity. These cells are present throughout the connective tissue of the body. A limited amount of data reveal that degranulation of mast cells and eosinophilic infiltration can be induced by exposure to phthalates [44]. Triclosan, found in cosmetics and personal care products, has been shown to inhibit RBL-2H3 mast cells by decreasing the mitochondrial membrane potential that leads to the inhibition of mitochondrial translocation and ultimately reduces the influx of calcium ions into the cells [45]. Researchers have shown that mast cells can be activated by BPA, resulting in accelerated release of histamines and leukotrienes [46].

Evidence on the impact of EDCs on mast cells implies that the alterations in inflammatory response and mitochondrial function can influence multiple cellular processes associated with inflammation, energy production, and utilization, ultimately, modulating energy homeostasis and leading to metabolic disruption.

EDC exposure also affects another group of inflammatory cells, neutrophils, the most abundant leukocytes in circulation. The impact of phthalates on neutrophils has been demonstrated by Vetrano et al. [47], who compared the effects of phthalate exposure on neonatal neutrophils and adult neutrophils. Neonatal neutrophils are susceptible to phthalate-mediated inhibition of (peroxisome proliferator-activated receptor-gamma (PPAR-γ), which ultimately leads to reduced anti-inflammatory signaling. Additionally, BPA induces neutrophil reactive oxygen species (ROS) production, which also alters neutrophil function by decreasing their chemotaxis ability [48]. An increase in ROS levels is directly associated with the inhibition of anti-oxidation activity, thereby causing oxidative stress [49]. Elevated ROS levels, oxidative stress, and reduced anti-inflammatory cytokines are crucial factors contributing to debilitating metabolic health and therefore, EDCs can stand as a significant factor in this epidemic scenario.

In addition to the aforementioned innate immune cell types, one effector lymphocyte class known as the natural killer (NK) cells are key instigators of the inflammatory response. They are the essential source of inflammatory cytokines such as TNF-α, IL-1β, and IFN-γ. An EDC, tributyltin, at the concentration of 5–100 nM enhances TNF-α production, whereas reduces its secretion at 200 nM [50]. Similarly, another EDC, dibutyltin, increases IL-6 production at a concentration of 0.05 and 0.1 μM but inhibits IL-6 production at 2.5–5 μM range [51]. These reports suggest that there is a differential effect on cytokine secretion with varying concentrations of EDC exposure. Therefore, we speculate that modulations in the cytokine pool can lead to inflammatory milieu, which is one of the hallmarks of metabolic disorder. 

Apart from the effects of EDC exposure during development on innate immunity, in utero EDC exposure has been recognized as a threat to metabolic health [52]. Maternal exposure to EDCs has been identified as a risk factor for unknown infertility and complications in pregnancy including preeclampsia, miscarriage, premature birth, and gestational disorders [53]. Schjenken et al. have shown that peripheral blood cytokine levels during the first trimester correlate with the presence of phthalates in urine, but they did not examine the effects of these changes in the babies [12]. Intriguingly, research by Kim et al. connected the presence of phthalates in the first urine sample of the newborn and the body mass index (BMI) of newborns after 3 months [54]; they found that exposure to phthalates (especially, diethyl phthalate) had a positive correlation with BMI surge. Another finding showed a positive association of prenatal urinary concentration of phthalate metabolites with the obese/overweight state of children (4–7 years of age) [55]. A recent study suggests that combinational exposure to BPA and high-fat diet perinatally contributes to cardiovascular and metabolic disorders in the offspring [56]. It is evident from the aforementioned studies that maternal exposure to EDCs can either result in obstacles during pregnancy and/or leave an imprint causing metabolic disease later in life.

In line with these findings, there is growing research interest in the possible impact of EDCs on immunomodulation of metabolic health. In this context, two broadly studied EDCs are phthalate and BPA [57]. Phthalate exposure in a hepatic cell line caused activation of the NLRP3 inflammasome, demonstrating a key role in liver damage that may lead to metabolic imbalance [58]. A recent study showed decreased adiponectin levels along with increased IL-6 and TNF-α secretion in human subcutaneous adipose tissue explants upon exposure to BPA [59]. Another study demonstrated that in 3T3-L1 preadipocytes, BPA stimulates JNK and NFκB pathways, enhances proinflammatory cytokine secretion, and causes insulin resistance [60]. It has also been recognized that EDCs may modulate the therapeutic potential of mesenchymal stem cells (MSCs) by affecting the differentiation potential and other biologic features including enhanced adipogenesis, reduced osteogenesis, elevated oxidative stress, and pro-inflammatory status [61]. In addition, EDCs reduce the immunomodulatory effect of MSCs [62,63,64]. However, the mechanisms through which immunomodulation is achieved by EDCs remain unknown. The effect of EDCs on MSC biology may be attributed to epigenetic modulation, which requires further exploration [61].

In summary, current evidence suggests that exposure to EDCs has an impact on the innate immune system. EDC exposure in utero or during development can result in immunomodulation, potentially leading to metabolic health defects in later life. There are various mechanisms of action of EDCs proposed so far; however, only a few reports suggest the role of EDCs in the immunomodulation of metabolic health via miRs. We will discuss this correlation in the next section. 

## 3. EDCs, MiR, and Innate Immunity

With rapidly developing high-throughput sequencing techniques, scientists have acknowledged that, even without protein-coding function, transcripts synthesized from a large portion of the genome have a pivotal role in many cellular processes [65]. Among other non-coding RNAs, miRs are short, single-stranded RNAs measuring around 19–25 nucleotides [66]. They are the most studied category in the non-coding RNA family [67], and have been shown to be significant regulators of mRNA [68]. They are indeed involved in many cellular functions related to energy metabolism [69,70] and are regarded as one of the key players in metabolic disorders [71]. 

Interestingly, the role of non-coding RNAs especially miRs in DOHaD gradually drew scientific attention in past decades [72,73,74]. As EDCs accumulate in our living environment and have extensive periods of biodegradation. Furthermore, long-term exposure can occur during the pregestational or gestational stages. Significant consequences on development can be observed in the next generation or across several generations [75]. Multiple sources suggest that the impact of EDC exposure on maternal and fetal miR profiles [76,77,78] is closely linked to the organ development of the offspring [79,80,81]. Studies have also suggested that dysregulation of the immunologic pathways is one of the key features of metabolic diseases, such as obesity and type 2 diabetes [82,83,84]. Furthermore, exposure to EDCs has also been found to induce metabolic disorders [85,86,87]. On the other hand, the role of miRs in regulation of inflammation-related genes, especially during the development of metabolic diseases, is described in many studies listed in Table 1. The above isolated findings show a significant correlation between miRs and EDCs induced immunomodulation during metabolic disease progression, as we have seen rapid growth in the number of studies focusing on the effect of EDCs on immunity via miR regulation [78,88].

### 3.1. Impact of EDC Exposure on MiR Regulation

EDCs, including both natural and synthetic chemicals, are initially found to induce adverse health consequences through interruption of hormone receptors. This includes estrogen receptors and retinoid receptors [9], especially during development, reproductive, and metabolic regulations [187,188]. Some of the EDCs can bind to the receptor proteins directly and can regulate downstream target gene expression [189,190]. Recently, studies have suggested other mechanisms on how EDCs induce the progression of the diseases. MiRs, which are susceptible to cellular stresses and environmental exposures [191], have been found to be influenced by EDCs as well. One of the insightful underlying mechanisms is that EDCs can regulate the miRs through their targeted hormones or receptors. Different studies in cell lines, rodents, and other vertebrates suggest that the expression of miRs can be regulated by hormones [192,193,194,195]. A study using MCF-7 breast cancer cell lines has shown that miR transcriptome alteration was induced by BPA and DDT treatment [97]. In another study, researchers showed that the treatment with perfluorooctanesulfonate alters the expression levels of about 38 miRs that are involved in thyroid hormone dysregulation [193]. A few studies also proposed that EDCs can directly modulate the expression level of miRs in different tissues or cell lines connected to metabolism [28,151,196,197,198]. For example, our recent study showed that the miR-34a-5p level is upregulated by benzyl butyl phthalate (BBP), thus promoting adipogenesis in 3T3-L1 cells through Nampt and sirtuins regulation [151]. Additionally, our data indicated that mono-(2-ethylhexyl) phthalate can induce regulation of several miRs, which are responsive to oxidative stress in vitro [28]. Besides the regulation of metabolism, EDCs also affect the cellular inflammatory response through miR modulation [196,197,199]. An in vitro study demonstrated that BPA treatment can induce the expression of miR-146a-5p, which mediates inflammatory response [197]. Lastly, tetrachlorodibenzo-p-dioxin, a pharmaceutical ligand, was found to induce cholinergic anti-inflammation through the upregulation of miR-132 [106]. One clinical study suggested that polychlorinated biphenyls (PCBs), which have similar structures as dioxin, induce miR-191 expression in peripheral blood mononuclear cells. Pathway analysis also identifies several potential targets of miR-191 that are associated with immunomodulation [113]. Taken together, EDCs may play a substantial role in alteration of miR levels and in the regulation of immune response that possibly contributes to chronic diseases such as obesity.

To understand the health consequences induced by EDCs, research focus has widened from their effect on one generation to their impact on the next generation as well as multiple generations. Since EDCs are persistent in our environment, their impression is more likely to pass through several generations and can even skip one generation and carry through to the next [200,201,202]. Moreover, when considering the prenatal and postnatal effects of EDC exposure, the situation becomes more complicated and is still under development. The transgenerational effects of EDCs on miR expression levels have been reported in several rodent and other mammal models [203,204,205,206]. For example, a mouse study showed genistein and/or BPA exposure at the developmental stage significantly affects the offspring’s miR expression patterns in the brain, leading to behavioral and metabolic alteration [203]. Paternal benzo[a]pyrene exposure led to miR expression pattern alteration in the offspring mice, and pathway analysis showed enriched target genes related to cell metabolism [204]. 

Several underlying mechanisms have demonstrated how miRs may navigate their effect in the next generation. First, the epigenetic imprint of miR levels can be inherited through the sperm cells [207], as they are sensitive to environmental factors such as EDCs [206,208]. Although there is no direct evidence to show that the miR profiles in oocytes is affected by the maternal environment, the effects of EDCs on DNA methylation and histone modification have been well studied in oocytes [209,210]. A mouse study showed that the oocyte transcriptome is affected in type I diabetic females [211], and alteration of miR levels is very likely to be one of the underlying mechanisms. 

Secondly, EDCs can act through modification of placental development to affect fetal intrauterine growth, although the mechanism is not fully understood [212]. One of the hormones that are significantly affected by EDCs, thyroid hormone, plays an important role in placental development [213,214]. Mounting evidence also shows that miRs regulate thyroid hormone signaling by targeting hormone synthesis and the expression level of its receptors [215]. On the other hand, miR expression can also be tuned by thyroid hormone [216,217]. In the mouse model, gestational DEHP exposure leads to thyroid hormone signaling disturbance and placenta malformation; this is accomplished through suppressing Thrα1 and Thrβ1 expression and their activity, ultimately causing fetal intrauterine growth restriction [218]. A cohort study also found that PCBs are associated with induced thyroid-stimulating hormone and thyroid hormone dysregulation in pregnant women [219]. This occurrence may lead to an adverse effect in the next generation, possibly through miR modulation. Other than that, in studies using in vivo or in vitro models, EDCs have been shown to affect other hormones such as human chorionic gonadotropin and corticotropin-releasing hormone along with impacting placental development through mechanisms including miR modulation [28,89,220,221,222]. It has been shown that exposure to BPA leads to miR-146a upregulation in both 3A and HTR-8 cells, potentially implicating its important role in inflammatory response and cell growth [89]. A study in the placental transcriptome also demonstrated that the miR-146a level is significantly upregulated and associated with BPA accumulation [223]. 

Lastly, Some EDCs can cross the placental barrier and reach the fetus, which would have a notable impact on its development [224,225,226]. EDCs can also be found in breast milk, while others are more likely to accumulate within the adipose tissue due to their hydrophobic nature [227]. These eventually provide prolonged adverse effects to those tissues. Due to this indirect exposure, fetal miR expression levels can be affected in different organs [203,205]. On the other hand, recent studies indicated that maternal miRs can traffic into the fetal side through the placental barrier [228]; this provides a new perspective of how the fetus can be affected by the external environment. These studies together implicate miR regulation in the transgenerational and postnatal effects of EDCs.

### 3.2. MiR in Innate Immunity

MiRs are expressed in various cells of the innate immune system such as monocytes, macrophages, dendritic cells, granulocytes, and NK cells [229]. They can rapidly respond to external stimuli [90]. Accumulating evidence indicates that miRs modulate immune responses at various steps of the innate immune network, including (1) production and release of cytokines/chemokines; (2) pattern recognition regulation; as well as (3) monocyte development and macrophage polarization. 

Inflammatory cytokines are a large family of secretory proteins, which play a key role in immune response regulation and metabolic homeostasis maintenance. Studies have suggested that miRs play an essential role in transcriptional regulation and secretion of cytokines [91,230,231,232,233]. For example, miR-155 was found to promote TNF-α expression by directly binding to the transcripts of its inhibitors in RAW cells [231]. TNF-α can also induce the expression of miR-155 [232], suggesting a reciprocal regulation between miRs and cytokines levels. Furthermore, miR-145 was found to promote TNF-α expression and secretion in human adipocytes [230]; it can also increase the bioactivity of TNF-α through inhibiting metalloprotease 17 [234]. Studies have also shown that miRs are involved in the determination of macrophage inflammatory response, which plays a critical role in obesity [235,236,237,238]. In a study using human monocytes, miR-146b was induced by LPS or IL-10, which led to the further downregulation of a cluster of Toll-like receptor (TLR) signaling genes, suggesting an anti-inflammatory role of miR-146b [237]. In another study, miR-497 treatment was found to inhibit the expression of pro-inflammatory cytokines, such as TNF-α and IL-1β, under in vivo or in vitro settings [238]. These studies suggest that miR regulation on cytokine secretion can further affect immune responses and metabolic health.

Monocytes and macrophages are predominantly derived from hematopoietic stem cells (HSCs) [239], playing an important role in inflammation regulation and homeostasis maintenance in different tissues [240,241,242]. Recently, miRs were found to be involved in the differentiation of bone marrow HSCs and the maturation of circulating monocytes [243,244]. A considerable array of miRs is highly expressed in the HSCs, such as miR-125, miR-126, and miR-146 [245,246,247,248]. Interestingly, overexpression of miR-125a has been shown to enlarge the hematopoietic stem cell pool by targeting pro-apoptotic protein BAK1 [249]. This has been shown to play a role in obesity and other metabolic diseases [250,251]. On the contrary, miR-126 serves as an inhibitor for stem cell proliferation [246]. Furthermore, the HSCs commitment to macrophage progenitors is also related to miR levels, especially controlled by the PU.1, a transcriptional factor. MiR-17p-92 is suppressed by PU.1 during myeloid differentiation, leading to the downregulation of a subset of miRs involved in myeloid progenitor maintenance [252]. These studies suggest a potential role of miRs in controlling major stages of monocyte development from HSCs. Importantly, the stemness and niche size of HSCs are sensitive to metabolic homeostasis and found to be disrupted in obesity along with other metabolic diseases [250,251,253,254]. Therefore, the maintenance of tissue homeostasis is likely to be connected to miR profile integrity and may be a factor in the metabolic scenario.

As we discussed at the beginning of the review, in metabolic tissues, such as the liver and adipose tissue, macrophages are key players in maintaining metabolic homeostasis [255]. Traditionally, macrophages are responsive to environmental cues that induce different pathways of cell differentiation to either M1 or M2. The phenotype associated with polarization towards M1 macrophages and secretion of pro-inflammatory cytokines is well characterized in many chronic diseases, such as obesity and nonalcoholic fatty liver disease [255]. Several reports suggested that miRs regulate either M1 or M2 polarization. For example, knockdown of miR-21 resulted in the reduction in M2 phenotype genes: arginase 1, mannose receptor 1, and IL-4Ra in vitro [256]. Furthermore, adipose tissue can produce or secrete a variety of pro-inflammatory and anti-inflammatory factors. This includes the adipokines leptin, adiponectin; resistin; as well as other cytokines and chemokines, such as TNF-α, IL-6, and MCP-1. These cytokines can stimulate macrophage polarization and produce negative feedback to the adipose tissue, therefore creating a vicious cycle [257,258]. Interestingly, in ewes, maternal obesity causes miR downregulation in the offspring’s muscle and further contributes to the upregulation of inflammatory cytokines [259]. Therefore, miRs can be an inevitable factor in the immune regulation and metabolic dysfunction. Overall, the interaction between miR profiles and inflammatory response has been extensively recognized and their roles in metabolic disease are significant.

In memoriam of Dr. Panzica, we would like to stress that EDCs also impact the neurological system as shown by his lifelong research. On this note, our understanding of miR functions is not limited to the metabolic processes mentioned above; it may also be expanded to include the modulation by miRs in other physiological activities [260,261,262,263]. A great number of studies indicate the tight connection between miRs and neural functions. A study showed that miR-219 was downregulated in a demyelinated mouse model via regulating MCT1 expression [264]. Another study on advanced paternal age (a risk factor for neurodevelopmental disorders) showed that miR-132 and miR-134 were both differentially regulated in rats and humans [265]. EDCs can affect the synthesis of various neuropeptides, hormones and enzymes, which results in an impact on the neural system and brain functions [266,267,268,269]. For example, Panzica group demonstrated that genistein affected the NO-producing cell number and induced significant changes in aggressive and anxiety behaviors in male mice [266]. In addition, whole-body metabolism and reproduction are also influenced by EDCs. Panzica group showed that postnatal genistein exposure induced body weight increase and significantly downregulated leptin and triiodothyronine, while the phenotypes were limited to female mice [267]. These studies suggested a potential mechanism of metabolism regulation by EDCs. Moreover, a study showed that expressions of several miRs in peripheral blood mononuclear cells were changed in anxiety disorder patients [270], suggesting a novel perspective link between miRs, immunity, and cognitive function. 

## 4. Conclusions

In conclusion, immunomodulation is one of the major events implicated in the progression of metabolic diseases where EDCs may play a significant role. Furthermore, several studies indicated miR as one of the mechanisms of action by which EDCs carry out immunomodulation in metabolic and immune tissues. The impact of EDCs on offspring miR levels is also substantial, leading to long-lasting modulation in the next generation. However, there is a lack of information on a comprehensive mechanism of metabolic disease caused by EDC exposure through the regulation of miR. Due to the unspecific nature of miR inhibition of mRNA translation, the complexity of miR profile alteration can be amplified while reflecting on the immune modulation and metabolic dysfunction. Further work is required to define the EDC-induced cellular and physiological sensing via miR in these tissues. Although direct and indirect effects of EDCs on miRs have been illustrated by a few in vivo, in vitro, or observational studies, more molecular regulations remain to be fully addressed by loss-of-function or gain-of-function animal models. Additionally, high throughput screening, big data analysis, and machine learning may provide critical facilitation of in-depth understanding of how immune systems are affected by EDCs through miR modulation and their contribution to metabolic disease progression.

## Figures and Tables

**Table 1:** MiRs affected by EDCs and their role in immune and metabolic processes.

EDCs	Primary Sources	MiRs	Role in Immunity	Role in Metabolic Health
Bisphenol-A	Industrial plasticizer	miR-146a	Inflammatory response and cell growth [89]; Inhibit inflammatory cytokines expression [90,91,92]; Inhibit inflammatory response through TLR-signaling [93]	Inhibit adipogenesis [94]; Protect β cell function [95]; Improve glucose metabolism and inhibit hepatic steatosis [96]
		miR-21	Tumor growth [97]; Promote liver inflammation [98,99]	Promote hepatic steatosis [99]; Inhibit triglyceride and cholesterol synthesis [100]; Promote adipose tissue browning and thermogenesis [101]
		miR-148	Inhibit IL-1β expression [102]; Improve immune dysfunction [103];	Inhibit proliferation and promote apoptosis [104]; Promote insulin expression [105]
Dioxin	Combustion	miR-132	Decrease IL-17, IFN-γ expression and suppress T-cell proliferation [106]; Enhance cholinergic anti-inflammatory reaction [107]; Upregulate NF-κB and STAT3 activity [107]	Promote β cell proliferation [108]; Inhibit neural tau expression [109]; Promote angiogenesis and endothelial proliferation [110]
Polychlorinated biphenyls	Dietary intake	miR-191	Inhibit inflammation by targeting DAPK1 [111]; Suppress inflammatory response [112]	Tumor growth [113]; Inhibit angiogenesis [114,115]; Induce DNA damage [116]; Block GLUT4 translocation and induce insulin resistance [117]
		miR-155	Regulate innate immunity [118,119]; Induce intestinal inflammation [120]; Inhibit the production of Th1-type cytokines [121]; Promote macrophage Inflammatory response [122,123,124];	Reduce oxidative stress and cell migration [125,126]; Promote adipocyte lipid accumulation [127]; Promote liver fibrosis [122]
Diethylstilbestrol	Birth control pills	miR-30	Promote M2 polarization [128]; Inhibit inflammatory response [129,130,131] Suppress humoral immune response induced by lipopolysaccharide [132]	Peripheral insulin sensitivity [133]; Promote apoptosis [134,135]; Promote adipocyte browning [136]; Inhibit adipocyte inflammation [137]; Regulate the intracellular lipid metabolism [138]; Regulate autophagy in thymocytes [139];
Methylparaben	Personal care products	miR-373	Targets the tumor-suppressor LATS2 and neutralize p53-mediated CDK inhibition [140,141]; Promote inflammatory response [142]	Inhibit proliferation and induce apoptosis [142,143,144]; Inhibit autophagy [145]; Inhibit liver steatosis [146]
Phthalate	Food sources	miR-34a	Regulate T cell function [147]; Promote inflammatory response [148,149,150]	Alter NAD levels and sirtuins activity Promote adipogenesis [151]; Inhibit glycolysis [152,153]; Promote mitochondrial respiration [152]
		miR-16	Inhibit B cell chemotaxis [154]; Promote inflammatory response [155]; Inhibit inflammatory response [156,157,158,159]	Promote apoptosis [27,160];
		miR-126	Inhibit T cell differentiation [161,162]; Promote inflammatory response [163]	Promote oxidative stress [28] Promote autophagy [164,165]; Promote lipid accumulation [164];
		miR-17	Inhibit inflammatory response [166,167,168]; Regulate B cell development [169,170]	Promote adipocyte browning [171]; Enhance insulin sensitivity [172]; Promote angiogenesis [173]
		miR-200c	Inhibit inflammatory response [174]; Promote inflammatory response [175,176]	Promote lipid accumulation [177]; Promote lipolysis [178]; Inhibit proliferation [179]; Enhance insulin sensitivity [178]
Triclosan	Personal care products, Cosmetics	miR-10a	Promote inflammatory response [180,181]; Inhibit inflammatory cytokine secretion [182]; Suppress M1 polarization [183]	Inhibit proliferation [184,185]; Promote adipocyte browning [183]; Prevent β cell degeneration [186]
